# Hybridized-GNSS Approaches to Train Positioning: Challenges and Open Issues on Uncertainty †

**DOI:** 10.3390/s20071885

**Published:** 2020-03-29

**Authors:** Susanna Spinsante, Cosimo Stallo

**Affiliations:** 1 Dipartimento di Ingegneria dell’Informazione, Università Politecnica delle Marche, Via Brecce Bianche 12, 60131 Ancona, Italy; 2 RadioLabs, Corso d’Italia, 00198 Rome, Italy; cosimo.stallo@radiolabs.it

**Keywords:** GNSS, autonomous railway traffic, uncertainty, accuracy, positioning error, 5G

## Abstract

In recent years, the development of advanced systems and applications has propelled the adoption of autonomous railway traffic and train positioning, with several ongoing initiatives and experimental testbeds aimed at proving the suitability and reliability of the Global Navigation Satellite System signals and services, in this specific application domain. To satisfy the strict safety and accuracy requirements aimed at assuring the position solution’s integrity, availability, accuracy and reliability, recent proposals suggest the hybridization of the Global Navigation Satellite System with other technologies. The integration with localization techniques that are expected to be available with the upcoming fifth generation mobile communication networks is among the most promising approaches. In this work, different approaches to the design of hybrid positioning solutions for the railway sector are examined, under the perspective of the uncertainty evaluation of the attained results and performance. In fact, the way the uncertainty associated to the positioning measurements performed by different studies is reported is often not consistent with the Guide to the Expression of Uncertainty in Measurement, and this makes it very difficult to fairly compare the different approaches in order to identify the best emerging solution. Under this perspective, the review provided by this work highlights a number of open issues that should drive future research activities in this field.

## 1. Introduction

Accurate radio-based positioning has recently boosted the exploitation of *location awareness*, i.e., the ability of a device to share its own physical location associated to a person or an object, in many different markets, spanning from Intelligent Traffic Systems (ITS) [1], to autonomous vehicles, Industry 4.0, and the improved management of communication networks [2].

**Figure 1:**
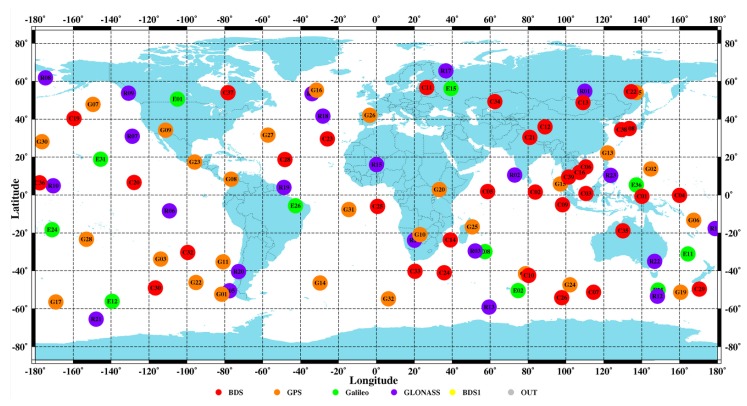
GNSS satellites’ positions at 23:00 on 31 December 2019 (from http://www.igmas.org, retrieved on 7 February 2020).

So-called Location Determination Systems (LDSs) encompass the necessary technological components to enable location awareness. Among them, the Global Navigation Satellite System (GNSS), a constellation of satellites transmitting positioning and timing data from space to GNSS receivers, that use these data to determine their location [3], still is the only LDS able to provide position, velocity, and time (PVT) necessary for localization purposes, on a global scale and on a continuous time basis. By definition, GNSS provides a global coverage, ensured by the harmonization of the technical specifications (such as frequencies and bandwidth allocation) among the European Galileo, the NAVSTAR Global Positioning System (GPS) from USA, the Russian GLONASS, and the BeiDou Navigation Satellite System (BDS) from China [4]. As of January 2019, there were 115 available GNSS satellites, with 32 GPS satellites, 25 GLONASS satellites, 34 BDS satellites, and 24 Galileo satellites. The GNSS satellites’ positions at 23:00 on 31 December 2019 is shown in Figure 1.

Galileo is the European contribution to GNSS, which, differently from other systems, is under civilian control and has been designed in response to the diverse needs of different user communities. Galileo supports four services, namely Open Service, Commercial Service, Search and Rescue, and Public Regulated Service, with different levels of position information accuracy, robustness, authentication, and security. The fully operational system foresees 30 satellites emitting a coded radio signal. A Galileo receiver computes its position calculating latitude, longitude, and time based on signals received from four satellites. Galileo-enabled mobile terminals feature an accurate and ubiquitous GNSS localization, with an accuracy within 1 m for professional users and 5 m for the general public. Such a relevant accuracy performance from Galileo is made possible by a number of technical improvements, compared to GPS and GLONASS, such as a stronger signals’ robustness to multipath, with a code phase error of ranging signals much lower than GPS, and the three orbital planes inclination enabling better Earth coverage at high latitudes. When all the 30 Galileo satellites are deployed and operational, six to eight satellites will always be visible from most locations, allowing positions and timing to be determined very accurately, down to a few centimeters. The reliability of Galileo services, and GNSS in general, is further increased thanks to the interoperability with GPS satellites.

Accurate and reliable positioning information provided by GNSS is nowadays exploited not only in aviation and autonomous cars or trains, but also in contemporary marine vessels, which are equipped with complex navigation and communication systems such as Electronic Chart Display & Information System (ECDIS), Automatic Identification System (AIS), Global Maritime Distress & Safety System (GMDSS), Integrated Navigation Systems (INS). GNSS PVT data are exploited by all of them, to assess the integrity of the positioning information, and mitigate interference, jamming, and spoofing in marine navigation [5]. In the majority of merchant ships, shipborne autonomous GPS receivers are still the primary means for positioning. More advanced GNSS receivers and satellite-based augmentation systems (SBAS) are still rare, but expected to become more common in the near future. Existing requirements, performance standards, and future concepts of maritime GNSS integrity are discussed in [6].

In autonomous cars and vehicles, several target Key Performance Indicators (KPIs) are set, which need to be strictly satisfied [1]. For example, a position accuracy < 20 cm is required by safety-critical automated driving [7]. Safety requirements are as relevant as those related to accuracy, for both autonomous trains and cars [8], and this has an impact on the requested integrity of the position solution. The per-hour Tolerable Hazard Rate (THR), defined as the occurrence rate the vehicle control systems fails to stop the vehicle at the desired location, or its speed exceeds the prescribed value, is used to quantify the safety requirements. In railway systems, an overall THR better than $10^{-9}$/h or even $10^{-10}$/h is mandatory, and this can be attained only through a cross-check with a GNSS-independent LDS, as shown by Lo et al. [9].

At present, the use of GNSS in European rail is primarily for non-Safety of Life (SoL) applications, such as asset management and passenger information services [10].

As a matter of fact, GNSS positioning still lacks the requested accuracy and continuous availability [11] in complex propagation environments such as high-speed moving trains, dense urban scenarios, tunnels, or multistory car parks, despite its global geographical coverage. In the aforementioned cases, augmentation of the vehicle localization capability by satellite-independent systems is necessary. The latest technological developments show that augmented GNSS, together with specific sensors, can help compliance to the stringent requirements associated to critical scenarios (railway LDSs or high-speed train positioning) for which the Comité Européen de Normalisation Électrotechnique (CENELEC) requires to fulfill the Safety Integrity Level (SIL) 4 [12].

In the Moving Block Signaling (MBS) system designed to efficiently improve the operation of railway lines, the moving authority of each train is calculated from the real-time positioning information of the preceding one [13]. A moving block (MB), defined as a virtual zone surrounding the train and dynamically determined from its position and velocity data, operates as a safety buffer inside which the train resides: other trains are forbidden to enter, and each train cannot leave its own safety buffer. To ensure safety of train traffic managed by the MB principle, a continuous, accurate, and reliable train positioning service is mandatory. It is actually a nontrivial work to develop a reliable MBS system for railways because of the requirements of highly accurate positioning, high capacity communications, and high performance separation control. Actually, there is no high-speed railway line operating in MBS mode in the world; instead, the Fixed Block Signaling (FBS) system mode is widely used because of its relatively high-safety property.

To increase the adoption of satellite-based positioning for railway signaling, the European GNSS (E-GNSS) Agency is working together with rail and space industry stakeholders. Their joint efforts led to the European Train Control System (ETCS), now being adopted both in Europe and beyond, as one of the components of the European Rail Traffic Management System (ERTMS). In the ETCS framework, nowadays the positioning of a train is based on balises, i.e., physical elements mounted at specific intervals along the railway track. Wherever possible, physical balises should be replaced by virtual ones (VBs), based on precise GNSS positioning, without any operational or safety implications on the ETCS. The main projects carried out to achieve an E-GNSS enabled ETCS, are presented in the related publicly available roadmap [14].

The discussion presented above shows that GNSS-based positioning still suffers several limitations with respect to the requirements set for reliable autonomous railway traffic, which could be tackled by hybridization techniques aimed at improving and augmenting the positioning data provided by GNSS. Among the proposed approaches, the integration of GNSS with localization techniques enabled by the fifth generation (5G) mobile communication is specifically targeted. Positioning will play a key role in the next 5G communication systems [15]: network pervasiveness due to densely deployed access nodes (ANs) will support precise location with enhanced availability, exploiting the network control links and signals in all kinds of environments, especially urban canyons, where most GNSS signals are blocked or affected by severe multipath. In those cases, unavailable or corrupted GNSS observables may be replaced by 5G observations added into GNSS solutions.

Several approaches have been proposed in the literature, but a direct comparison or evaluation among them is quite difficult to carry out. In fact, the overview of the recent state-of-the-art proposals for GNSS hybridization provided in this work, under the perspective of the related uncertainty issues and attained performance [16], shows that in many cases the declaration of the uncertainty in the positioning measurements attained by different studies is not consistent with the Guide to the Expression of Uncertainty in Measurement (GUM) [17]. It is consequently difficult to assess the different proposals and identify the best-performing one. The review carried out in this work aims to highlight such a limit in the currently available scientific and technical data resulting from different experiments and testbeds, in order to raise awareness about the need for standardized approaches that could enable a fair benchmarking. To the authors’ best knowledge, such a kind of analysis is not yet found in the literature.

This work is organized as follows. Section 2 reviews the main measurement techniques used in wireless positioning systems, focusing on GNSS positioning. The most recent state-of-the-art proposals for hybridized-GNSS systems applied to train positioning are presented in Section 3, mentioning a recently appeared 5G-only approach too. The selected literature is analyzed under the uncertainty perspective and with respect to the results obtained in Section 4, while the limits of the methodologies adopted in the different studies are discussed with respect to the requirements prescribed in railway traffic in Section 5. Finally, Section 6 concludes the paper and provides some insights on future trends based on currently ongoing research initiatives.

## 2. Wireless Positioning Systems

Wireless positioning systems encompass either nodes with known locations and nodes whose position has to be determined. They are identified as *known nodes* and *unknown nodes*, respectively. The former may be at fixed locations, such as anchor nodes in local positioning systems, or not, as it happens in satellite positioning systems to provide a global coverage. The latter may be stationary or mobile nodes, as in navigation systems.

### 2.1. Measurement Techniques

The conventional approach to estimate the location of an unknown node relies on a two-steps method: first, measurements are derived from the received radio waves’ characteristics; then, the position of the node is estimated by means of different calculations performed on the measurements taken. The last ones include, among others: (i) the difference in the power of the Received Signal Strength Indicator (RSSI), compared to the original signal strength; (ii) the Time of Arrival (ToA) and Time Difference of Arrival (TDoA); and (iii) the angle of Arrival (AoA) of the signal. A set of measurements of the same kind (AoA, RSSI, or ToA) is used by some position estimation approaches to establish the location of an unknown node; other solutions combine AoA information to distance estimates derived from RSSI or ToA.

Table 1 summarizes the main wireless positioning systems with their corresponding measurement techniques (elaborated from [18]). Although localization has long been considered an optional feature in the framework of standardization, implementation, and exploitation of existing cellular networks, the global cellular communication infrastructure deployed around the world can actually support positioning services. Several contributions have been provided by the research community to the development of positioning strategies within each generation of cellular technology, from the first one (1G) to the upcoming 5G. As appears in the table, different measurement techniques have been adopted in mobile cellular systems, according to the advent of different mobile networks generations.

The approximate location of a user’s device, relative to the cells of the mobile communication network, is identified by a numerical parameter called Cell ID, in those cellular systems where local positioning is supported. For third-generation (3G) mobile terminals, the Enhanced Observed Time Difference (E-OTD) is a standard localization method, based on time difference measurements performed in the handset rather than the network, and a mechanism to pseudo-synchronize the network [19]. In 4G Long Term Evolution (LTE) mobile networks, the Observed Time Difference of Arrival (OTDoA) is used to determine the position of User Equipments (UEs) [20,21]. Multilateration is exploited in the Uplink-Time Difference of Arrival (U-TDoA) [22], based on the accurate measurement of the time it takes a signal to travel from a mobile phone to multiple sensitive receivers, called Location Measurement Units (LMUs). No specific chip onboard the UE is needed, as computations are performed by the network, thus this technique is available also to older generation mobile terminals.

Most of the cellular networks only provide basic localization methods and assistance data for GNSS, essentially because of the increased costs network operators should otherwise face. 3GPP LTE (4.5G) includes potential enhancements for positioning technologies which are both depending on Radio Access Technology (RAT), and RAT-independent, as summarized below [23]:

- OTDoA enhancement based on more density in the time domain, a new pattern and an extension of the Positioning Reference Signal bandwidth enabled by Carrier Aggregation, even combined with cell-specific reference signal and transmitted in unlicensed bands (LTE-U);
- Device-to-Device aided positioning: user equipments with a known position (acting as anchored nodes) could cooperate with target equipments to improve their location accuracy, exploiting the location of neighbour devices or using ranging and signal strength measurements;
- Multiple Input Multiple Output (MIMO): multiple antennas and beamforming techniques can improve the vertical positioning of the user equipment;
- WLAN/Bluetooth: ranging and signal strength from WLAN and Bluetooth networks could be combined with LTE-based measurements for positioning, exploiting inter-RAT functionalities;
- Terrestrial Beacon Systems: beacon and Positioning Reference signals could be transmitted with a dedicated infrastructure to enhance the network-supported positioning capabilities; and
- Barometer: barometric sensors embedded in user equipments can improve the accuracy of vertical positioning.

In Release 13 of TS 36.305 [24], the accuracy requirements for ranging or Reference Signal Time Difference (RSTD) measurements were updated, according to the use of received signals at the same carrier frequency (intra-frequency measurements) or at different carrier frequencies (inter-frequency measurements), as shown in Table II of [23]. As discussed in [25], 5G networks aim for sub-meter accuracy in 95% of served areas, comprising both indoor and urban environments, thus overcoming by at least one order of magnitude the accuracy level of the current state-of-the-art solutions, including GNSS in non-Line Of Sight (LOS).

### 2.2. GNSS Positioning

**Figure 2:**
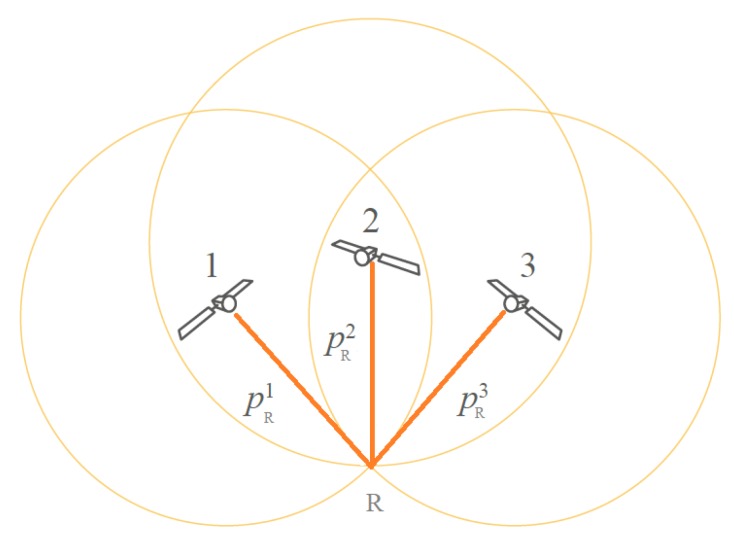
Receiver’s pseudoranges ($p_{R}^{i}$, i=1,2,3) from three satellites.

A GNSS receiver collects signals from the GNSS satellites that in a given moment are located in its field of view, as shown in Figure 2. By applying correlation techniques on the received signals, the receiver measures the so-called *pseudoranges* from each satellite. These measurements are affected by various sources of uncertainty, such as variations in the measured signals, due to multipath. Sub-metric position accuracy is attainable only by mitigating as much as possible the different sources of uncertainty. An additional fixed GNSS receiver with a precisely determined position is used in many techniques. Such a receiver acts as a *reference station*: it collects GNSS signals similar to a classical receiver, but, based on its exactly known position, the corrections to be applied on the pseudoranges may be computed. They allow obtaining sub-metric accuracy when the GNSS receiver is placed in an open-sky environment, not subject to strong multipath.

Multipath originates from the combination of LOS and Non-LOS (NLOS) signal components, due to reflections off surroundings before reaching the receiver’s antenna. In the presence of multipath, the code tracking procedure performed by the receiver cannot correctly distinguish the actual correlation peak between the received signal and the locally generated replica at the receiver, with code phases errors up to tens of meters. In GNSS, the available redundant measurements due to the presence of many satellites in the receiver’s field of view allow implementing a specific procedure to mitigate multipath, which is based on detecting and excluding the affected measurements. This procedure is known as de-weighting [26,27]: suitable algorithms are needed to detect and de-weight distorted signals, at the same time avoiding significant degradation in the measurement geometry. In fact, a poor geometry may even exacerbate the effects of remaining errors and worsen the ultimate position solution. The incidence of geometry degradation may be estimated by a metric called weighted Dilution of Precision (DOP).

Precise Point Positioning (PPP) [28] and Differential GNSS (DGNSS) [29] are the two coexisting methods to generate and provide range corrections. Then, two main DGNSS approaches exist: standard and Real Time Kinematic (RTK), depending on the type of measurement used. In standard DGNSS, the user’s receiver computes its position by applying algorithms based on GNSS signals’ code measurements from the reference station and the receiver itself. RTK algorithms, on the contrary, are based on carrier phase measurements. Decimeter level accuracy may be attained by PPP technique in kinematic mode; centimeter level or better is possible in static mode, thanks to precise orbit, clock, and error models. When GPS is the only constellation used for DGNSS (which is then called DGPS), accuracy is in the order of 1 m for users located in the range of few 10s km from the reference station, growing (i.e., worsening the quality of the positioning information) at the rate of 1 m every 150 km of separation. RTK DGNSS reaches centimeter-level positioning accuracy.

## 3. Hybridized GNSS for Train Positioning

When GNSS will be able to ensure the same safety level achieved by traditional systems, the European Railway Traffic Management System/European Train Control System (ERTMS/ETCS) will experience a disruptive innovation. To this aim, augmentation solutions, and integrity monitoring techniques are needed. An additional system, usually a terrestrial one, can cooperate to improve the positioning performance of a satellite navigation system, through augmentation. In Assisted GPS (A-GPS), i.e., GPS augmented with a cellular network, the burden of position computation is shifted from the receiver to the network, which makes the information available to the UEs within the cell.

In [30], two approaches to train positioning are compared, namely the DGNSS and the code double difference, both featuring an external augmentation to GNSS signals. Based on the assumption that the train is constrained to the track during its ride (so-called track constraint imposition), the train position is derived as the curvilinear abscissa of the track where the reference point of the train is lying on. The difference between the two approaches relies in the way the curvilinear abscissa is used to replace unknowns in the mathematical models used for positioning computation, both based on the extended Kalman filter (EKF) to solve the resulting equation set. The survey by Otegui et al. [31] analyzes the main train positioning solutions currently in use, looking at common parameters and criteria, such as applied sensors and algorithms, validation tests, results obtained. Sensors typically used in railway positioning include onboard Doppler radar, wheel sensor (called tachometer or odometer) and balise transponder. Recent studies have shown that Eddy current sensors can be used as speed sensors. The position information provided by fixed external balises, gathered by the train balise transponder, is currently exploited to compensate the long-term drift of the onboard odometry device. Ongoing research activities aim to replace the external balises with a Virtual Balise Reader (VBR) based on GNSS and embedded in the train itself, to periodically get positioning data sampled at higher frequency. A GNSS-based VBR would functionally operate as a physical balise, or a balise group. The last ones would still be necessary in situations where the VB cannot be available, such as in tunnels, or where GNSS provides poor performance. Environmental conditions and local effects [12] can increase the uncertainty of the GNSS-measured position, making it unacceptable with respect to user requirements [32].

It is well-known that *pseudoranges* measured by a GNSS receiver in a VBR are affected by various sources of uncertainty:

- Satellite clock: Even if the timing equipment of GNSS satellites is very precise and corrections are broadcasted in the GNSS signal, a small clock bias remains. In the downlink data, the satellite provides the user with an estimate of its clock offset, which gives a typical uncertainty of about ±2 m, although this value can vary between different GNSS systems.
- Satellite ephemeris: A GNSS satellite broadcasts its own position within the signal, but, even with the corrections from the GNSS ground control system, small errors in the orbit can result in up to ±2.5 m uncertainty.
- Ionosphere and troposphere: Ionospheric activity can delay signals in the GNSS frequency bands, causing a significant uncertainty in satellite position, typically much greater than ±5 m. Variations in tropospheric delay are caused by the changing humidity, temperature, and atmospheric pressure in the troposphere, but they are very similar on a local scale, so that their effects can be compensated by DGNSS or RTK techniques.
- Multipath: GNSS signals are reflected and delayed by ground infrastructures and vegetation, resulting in a typical position uncertainty of ±2 m.
- Receiver noise: It originates in the thermal noise generated by the receiver RF, providing a typical ±0.1 m uncertainty.
- Receiver clock: Mass-market receivers contain oscillators cheaper than the ones on-board satellites. However, because all GNSS signals are similarly affected by the receiver clock, this error is easily mitigated by the receiver during the computation of the position.

The above sources of uncertainty are summarized in Table 2.

PPP techniques may mitigate the first three contributions, while multipath cannot be compensated by a reference station, being a very local source of error. The receiver noise cannot be corrected either, being specific to each GNSS receiver. Hybridization of GNSS with one or several other localization techniques in an integrated navigation solution can overcome these issues: by adding devices or signals, position information can be provided on a continuous basis, even when satellite signals are not available, such as in tunnels. The fusion of GNSS and ten Degree Of Freedom (DOF) Inertial Measurement Unit (IMU) is evaluated in [33], for train positioning. The proposed method to calculate train velocity is based on the raw measurements provided by the GNSS receiver and the IMU, used to compute track features. They are then compared to a reference velocity obtained from tachometer and Doppler radar readings. Field measurements obtained in the study show that, even with a simple data fusion algorithm, the calculated velocity approaches the one returned by higher cost sensors, such as Doppler radars typically available onboard a train. However, in the case of a GNSS signal outage, additional information is required to guarantee the validity of the solution.

Due to environmental limits, service availability of satellite signals, which is essential for field applications of satellite positioning in train operation condition monitoring and safety control, cannot be guaranteed completely. Satellite positioning availability can be improved through multi-sensor information fusion, used to compensate signal shadowing in constrained operating areas, such as railway stations with canopy. To this aim, the so-called pseudolite (PL) technology makes it possible to enhance the GNSS availability: even when the true GNSS signals are completely blocked, thanks to a pseudolite signal similar to the one radiated by navigation satellites, the receiver can still perform the navigation calculation [34]. It is not a true hybridization approach, but it may be mentioned among the sensor fusion-based solutions. In [35], a method to optimize the pseudolite constellation design in railway stations with a constrained GNSS visibility condition is presented, considering both a degraded GNSS observing scenario, and a fully GNSS-denied operating scenario, the latter taking place when the train is moving in the canopy-covered track area of the station. The Performance Indication Factor (PIF) is used as a metric to assess the performance of the satellite constellation, considering both GNSS and PL, and aiming for its minimization. The paper shows how, in the degraded GNSS scenario, the PIF from the hybrid GNSS/PL mode is lower than that from the GNSS-alone mode. Such a result is attained involving pseudolites in the station area, joint an optimization strategy for their enhanced placement. The paper does not report results in terms of positioning accuracy, because performance is given in terms of PIF that is found to be, in some conditions, relatively high, thus showing that the adopted pseudolite constellation fails to provide the expected locating enhancement capabilities.

Minetto et al. [36] provided a comparison between the legacy Extended Kalman Filter (EKF) and a suboptimal Particle Filter (s-PF) within an integration paradigm aimed at the collaborative hybridization of independent heterogeneous measurements, for enhanced vehicular localization capabilities. The proposed framework relies on the exchange of navigation data and positioning solutions among GNSS receivers through an ad-hoc network, which supports the combination of GNSS observable measurements. In a non-collaborative framework, s-PF easily overcomes EKF performances at the cost of a higher computational cost, in a realistic scenario, just providing network connectivity among few GNSS receivers, allows a hybridized EKF implementation to reach and improve over s-PF outcomes. The paper shows that EKF can be enhanced by integrating auxiliary correlated information more efficiently than adding more particles in s-PF implementation, for which the attainable average accuracy improvement decreases from 23.02% in good visibility to 12.36% in poor visibility conditions. It is important to point out that all the results are obtained by simulations, and no field experiments in a real scenario have been performed.

The 5G-CHAMPION project [37] proposed a GNSS and 5G integrated positioning solution to overcome both insufficient satellite availability and multipath in harsh environments. Pseudoranges from GNSS are integrated by angular information provided by the 5G network, in order to lower the number of GNSS satellites required to compute a position. GNSS signals affected by multipath can be removed from computation, as the 5G Line of Sight (LoS) information provides extra equations to solve the positioning problem. In [38], the proposed GNSS/5G hybridization consists in adding 5G observations in the measurement vector of both single and PPP GNSS, again by exploiting the angular information from 5G. An accuracy in the order of 1 m or even below is expected for positioning supported by 5G networks; additionally, Werner et al. [39] showed that positioning algorithms can run at the network side, ensuring a highly energy efficient approach from the UE’s perspective.

In the connected vehicle scenario, for which 5G will act as a fundamental enabler, technical solutions that facilitate obtaining and providing device location information with both high-accuracy and low power consumption at the UE are possible [40]. Simulation results indicate achievable localization accuracies below ±30 cm by using cellular ranging measurements with 50 and 100 MHz of system bandwidth. A hybrid positioning solution combining 5G and GNSS is presented in [41], based on a two-step approach to tightly couple the two technologies. The authors assumed a UE connected to a 5G base station and able to receive a number of GNSS satellites, thanks to an antenna array to serving both 5G and GNSS signals. In the theoretical study, the Fisher Information Matrix (FIM) of a hybrid 5G-GNSS localization estimation is derived, as well as the position and rotation error lower bound. Results are obtained through simulations only, and they indicate that beyond a certain value of the carrier-to-noise signal ratio (CNR), a practical 5G positioning can improve its accuracy through GNSS integration. The value of such a CNR threshold depends on the number of visible satellites. The authors stated that a specific positioning requirement of 50 cm can be achieved by a very favorable GNSS-only configuration (five visible satellites and high CNR), or, alternatively, by the hybrid solution with three visible GNSS satellites, lower GNSS CNR but good 5G signal-to-noise ratio.

The use of mmWave technology is one of 5G communications’ key aspects: it enables high data rates and beamforming [42] for AoA positioning, through the use of a large number of elements on antenna arrays for massive multiple-input-multiple-output (MIMO) systems. In [43], high-speed train positioning using specific, beamformed 5G New Radio (NR) downlink synchronization signals (SS) is investigated, based on the sub-meter positioning accuracy requirements specified by the 3GPP Release 14. The Primary Synchronization Signal (PSS), the Secondary Synchronization Signal (SSS), and the Physical Broadcast Channel (PBCH) are transmitted by a network of fixed Remote Radio Heads (RRHs): this ensures very good built-in correlation properties, resulting in accurate ToA and Angle of Departure (AoD) measurements. However, the good results obtained from simulations do not yet satisfy mission-critical requirements, thus requiring an appropriate fusion with GNSS measurements. Despite being challenging to deploy and expensive, the envisioned architecture could be an effective replacement of the obsolete one currently in use, i.e., the international wireless communications system for railway communication and applications (GSM-R).

## 4. Results analysis

To qualify the result of a measurement activity, its associated uncertainty must be provided [17]. This section briefly recalls the results presented in the studies analyzed before, highlighting the way uncertainty has been expressed.

Two GNSS augmentation approaches tested in [30] provide similar results in terms of positioning *error*, compared to a ground truth position information obtained by means of an external RTK service. A car travelling on a highway instrumented with an ad-hoc augmentation network emulates a train ride. Either in differential or double difference mode, the differences between measured and ground truth position exhibit a Gaussian distribution, from which a ±1 m standard uncertainty at 99% confidentiality level arises. In this case, as prescribed by the GUM, the statistical distribution of the measurements collected in the experiments is determined and, based on that, the standard uncertainty with associated confidence is provided. The double difference mode declares four epochs of system unavailability, against the less conservative differential one. The former has the advantage of removing the clock issues from the navigation solution.

The performances of a local augmentation and integrity monitoring network were analyzed in a previous work by some of the authors [44], where a standard uncertainty of ±2 m for a Gaussian probability distribution of the differences between the measured and the ground truth positions is presented. Except for some cases in which the standard uncertainty increases to ±3 m, the declared confidence level is 99%. Having adopted the same modality to express either the experimental outcomes or the measurement uncertainty, based on a Gaussian distribution in [30,44], the results obtained may be fairly compared.

A real setting, with three there and back train rides between two stations, for a total covered distance of 54.53 km, has been arranged to test the fusion of GNSS and 10 DOF IMU measurements in providing train velocity data in [33]. The presence of tunnels along the railway determined GNSS signal outages, whose time duration was varied by modulating the train’s velocity. Reference values of train velocity were obtained by synchronized tachometer and Doppler radar readings. According to the results detailed in the paper, the uncertainty in estimating the train velocity was within ±2 km/h with a 93.53% probability, and within ±1 km/h with 80.90% probability, when the drift in IMU-only measurements (due to GNSS outages) were excluded. Taking into account the GNSS outages, the resulting uncertainty was within ±2 km/h and ±1 km/h with a 88.90% and a 76.85% probability, respectively. In this study, the attained results are expressed in terms of estimated train velocity, and not in terms of train positioning error, as specified in the works analyzed above. As a consequence, the direct comparison among the different approaches is not possible. Additionally, the statistical distribution of the attained measurements is not specified, whereas it would have been important to characterize the influence of GNSS signal outages on the resulting uncertainty.

The fusion of GNSS signals with other sensory data was also discussed by Wu et al. [45], who evaluated the reliability of a GNSS-based train localization unit that exploits measurements from different sensors and a Petri Net to attain localization information. The train localization unit includes a GNSS receiver, an Inertial Navigation System (INS), and a data processing unit (DPU): the INS provides original localization determination, with position and velocity based on the output of internal gyroscope and accelerometer, while GNSS offers the pseudorange and pseudorange rate. A Kalman Filter (KF) corrects the localization determination and the output of the INS. The technical specifications of the INS and GNSS components are provided in the paper. The evaluation of the train localization unit is performed on field test data collected from the Beijing–Shenyang railway in August 2018. The reference system used simultaneously with the unit under test is a NovAtel SPAN-FSAS GNSS/INS receiver, featuring a centimeter-level positioning. The analysis of the experimental data shows that, if the localization unit uses INS alone for about 10 s, the positioning offset with respect to the reference system reaches about 20 m, which cannot satisfy some localization-based applications in railway. Adding GNSS data, the attained failure-rate of the localization is not low but there is room for further improvement. This study provides results in terms of positioning *offset* that can be related to the positioning *error* mentioned in [30,44]; however, details about the qualifying uncertainty are not provided.

In [38], the performances of hybrid GNSS and 5G positioning are assessed in a field test-bed with clear-sky, urban and canyon GNSS visibility conditions. The L1 band only is used to collect GNSS observations, as it mostly happens with the operational capabilities of current mass market receivers, whereas 5G observations are provided through a Transmission Control Protocol (TCP) stream. The 5G conditions are described by the relative position of the 5G base stations (BS) and the UE: 20 m to the north (N20); 20 m to the north and 20 m to the east (N20 E20); and 20 m to the north and 20 m to the southeast (N20 SE20). The same locations are also tested at a distance of 50 m. In both cases, the uncertainty of the BS-to-UE distance is not reported. A geo-referenced position in the open-sky environment provides the ground truth, whereas a precisely computed position is used for the urban and canyon settings. According to the way results are reported (Table 1 in [38]), the following outcomes hold: in the clear sky setting, the authors reported a best mean accuracy of 0.320 m at 99th percentile in (N20 E20) configuration with PPP and 5G; in urban scenario, best mean accuracy of 0.918 m is reported in (N20 E20) with 5G only; and, finally, in canyon scenario, the best mean accuracy declared is 0.7272 m in (N20 E20), using 5G only. 5G is especially important in the urban environments where GNSS signals are less available and the base stations are close enough to the UE.

High-speed train positioning using 5G only is simulated in [43], where a track of over 43 km is modeled, with variable train velocity of up to 400 km/h. Using channel models suitable for the considered scenario, and AoD and ToA measurements with the EKF-based tracking model, the authors declare a mean positioning accuracy of 0.66 m (below the 1 m 5G target), with 95% errors below 1.7 m, and 99% ones below 2.3 m. Separate PSS/SSS sequence identities are assigned to RRHs uniformly located at 500 m intervals along the upper side of the railroad, at a distance of 15 m from the railroad. This way, the same combination of PSS and SSS sequences cannot be heard in the same location from multiple RRHs, and the PSS sequences of adjacent RRHs are always different. Despite the promising outcomes, it is observed that, whenever the train acceleration changes, the tracking algorithm introduces a lag, which affects the tracking accuracy over a certain period. In the works by both Maymo-Camps et al. [38] and Talvitie et al. [43], the attained performances are expressed in terms of mean positioning accuracy; however, details about the statistical distribution of the measurements are not provided, and the coverage factor used to compute the accuracy is not specified as well. Additionally, despite being based on the fusion of different technologies, i.e., GNSS and 5G, the former study does not analyze the uncertainty propagation associated to the combination of different quantities to derive the position information.

## 5. Discussion

In the discussion of solutions based on GNSS measurements hybridization (i.e., fusion) with other measurements of different nature, it is important to recall the fundamental benefit motivating such approach, i.e., *reducing uncertainty* by combining information from multiple sources [46]. The results summarized in Table 3 motivate some considerations. First, performance comparison among different hybridization approaches is not straightforward. In fact, even if all the works considered aim for the same outcome, i.e., improving train positioning, it is evident how different studies evaluate their proposed approaches in diverse test-beds, obtaining different measures (either train positioning or velocity) whose uncertainty typically is not expressed according to the GUM. This way, it is not easy to say which hybridization approach performs better. Second, each study differs in theoretical foundation and performs different experiments or simulations, usually in environmental and operational settings that are in no way similar. Some studies rely on simulations only, others are supported by real-world experiments, but in conditions that are difficult to replicate, or cannot be fully controlled, e.g. outages, multipath, or fading effects due to the environment in which the train moves. Lastly, sensors used to test the proposed hybridization approaches are different from one study to another, and their own metrological characterization is usually missing.

According to the GUM, the measurement procedure shall be replicable and repeatable *under the same measurement conditions*. In real railway operation conditions, the positioning accuracy attained by the different studies shows strong variations; at the same time, the *dynamic* train localization accuracy is among one of the most relevant KPIs of the GNSS/hybridized-GNSS receiver, mandated by safety constraints in railway applications. As a consequence, the accuracy in both the along-track and cross-track directions of the train positioning should be evaluated under dynamic movement, possibly using bidirectional error models. However, measurements in which at least one of the quantities of interest is time-dependent, i.e., it changes on scales from picoseconds up to several minutes (called *dynamic measurements* [47]), pose additional challenges to the existing mathematical models and measurement methodologies. With the same motivations that steered the deployment of the *Galileo above* test bed (Galileo Application Center for Ground-Based Vehicles) in Germany in 2010 [48], different GNSS-hybridization approaches should be tested under identical, repeatable test conditions, which cannot happen if a pre-defined railway track is not used as a reference by all the experiments. In fact, the ISO 5725 [49] defines the repeatability limit as the value less than, or equal to which the absolute difference between two test results, obtained *under* the repeatability conditions, may be expected to be, with a probability of 95%. For measurements aimed at train localization by GNSS or hybridized-GNSS receivers, the experiment could be made twofold: static measurements at fixed antenna location, and dynamic measurements along a pre-defined railway track [50], with uncertainty expressed according to the GUM.

Considering the safety performances of the approaches analyzed in Table 3, the solutions presented in [30,44,51] report four epochs of system unavailability, and normal operation of the system for all the considered epochs, respectively. No details about the safety performances were provided by Otegui et al. [33] and Maymo-Camps et al. [38], whereas Talvitie et al. [43] reported a submeter accuracy with 75% availability, not enough for mission-critical use cases, such as autonomous trains. In [52], the authors studied and compared two fault detection methods for GNSS, based on an embedded odometer and on a single-axis accelerometer, respectively. The methods suffer from uncertainty on the accuracy of the track geometry information that is a necessary parameter for both of them, but this aspect is not further developed in the mentioned paper.

In the framework of the ERTMS evolution plan, GNSS is seen as a *game changer* innovation, with the potential to eliminate most of the track circuits and to increase the transport capacity, thus reducing the costs and contributing to a greener transport means. Moreover, the main goal of the introduction of the GNSS is the cost reduction without compromising the system safety. Considering that conventional LDS, such as those based on RF transponders (balises) deployed along the railway, are not economically sustainable for regional and freight lines, and that these lines constitute a big percentage of the European railways, the market slice addressed by such an innovation is quite huge. In fact, compliance with the safety requirements imposed by CENELEC norms is very challenging, so that adoption of an external AIMN (Augmentation and Integrity Monitoring Network) is highly recommended [9].

Concerning the availability, in the rail environment, the integration among the GNSS and a set of external sensors (e.g., mechanical odometers, inertial units or imaging sensors) can be exploited to increase system reliability and availability in GNSS denied areas (e.g., tunnels) as well as to improve system accuracy and integrity (e.g., by detecting and mitigating severe multipath or electromagnetic intentional and unintentional interferences).

From the operational point of view, uses of the train LDS include: (i) determination whether the train is in the proximity of those locations that may be trespassed only by authorized trains (this function is marked as VITAL in ERTMS); (ii) determination of the track on which the train is operating and its location on that track (function marked as NON-VITAL in the standard); (iii) detection of the eventual split of a train (train integrity assessment); and (iv) determination of the mutual distance among trains. Concerning Cases (i) and (ii), although different accuracy requirements to the LDS may be set to take into account the accuracy offered by today LDSs, the approach followed by the railway operators leading the introduction of the EGNSS in ERTMS is to define as target the same accuracy achieved by the conventional balise readers, as illustrated in Table 4.

This choice is the key to support full interoperability allowing trains equipped with next generation LDS to operate on lines equipped with physical balises, without the need of adopting different train control rules and procedures depending on the on-board equipment. Cases (iii) and (iv) are relevant for the upcoming ERTMS Level 3, so that the related requirements are still to be defined. Nevertheless, concerning performance, the driver is the decimeter accuracy required at least for the VITAL functions.

To reach the ambitious SIL-4 (Safety Integrity Level) target in terms of THR, the ERTMS has eliminated the need for control via light signals to drivers, eliminating the potential for human errors. The train is remotely controlled by the RBC (Radio Block Center), which periodically transmits to the train driver a proper authorization to proceed until the next location under speed supervision. If the train speed exceeds the authorized speed profile, the train is automatically stopped without human intervention. To satisfy the requirements reported in Table 4, data fusion of GNSS and other independent sensors is needed.

## 6. Conclusions and Future Perspectives

With the progresses in the deployment and near-term completion of Galileo, and thanks to the successful implementation and adoption of Europe’s space-based positioning augmentation system, EGNOS, the basis for a more widespread integration of satellite-based support in the railway sector is now laid. The question to address in the near future is not about what type of possible support GNSS can provide to the railway sector, but how such a support may substantiate. The research studies and experiments discussed in this work have shown that GNSS is able to make rail systems less complex, cheaper, more reliable and responsive, while maintaining the highest standards of safety, but the roll-out of space technology in the railways sector needs to be accelerated, by defining the future system architecture and quickly moving the solutions from the laboratory or pilot deployment onto the track. Interoperability and standardization are the preconditions for a true industrial innovation in the railway sector, which could bring additional advantages such as decarbonizing the transport system and increased sustainability. Satellite technologies are key to increasing capacity and efficiency in the system by enhancing quality and consistency of train localization, but all new rail systems must be certified and still there is an open issue about who should be responsible for such a certification. A similar discussion was carried out in the aviation sector, where the introduction of space technologies had a disruptive role as is expected for the railway transport system. Within the European Shift2Rail Joint Undertaking [53], the project GATE4Rail (GNSS Automated Virtualized Test Environment for RAIL) [54] aims to implement a geo-distributed simulation and verification platform connecting GNSS centers of excellence and ERTMS/ETCS laboratories, to evaluate the GNSS performances in the railway environment with agreed methodologies and tools. This way, it will be possible to stress the global system in presence of very rare fault events instead of long and expensive tests on field, thus developing a standard methodology for representative test cases based on a common test process framework, for zero on-site testing, to minimize deployment cost and time.

In this work, the main GNSS and hybridized-GNSS based techniques for automated railway traffic and train positioning proposed in the literature are reviewed and analyzed, with a specific focus on the issues related to the expression of the position location uncertainty and the measurements accuracy declared by the different studies.

The performed review highlights a lack of repeatable and comparable results, typically attained with different experiments and test-beds, as well as a number of different ways to express uncertainty, not always in line with the Guide to the Expression of Uncertainty in Measurement. Additional challenges to the existing measurement models and methodologies are posed by the dynamic nature of the positioning systems, and by the fact that the proposed approaches rely on the same satellite-based localization systems that should represent the reference against which the attainable accuracy must be evaluated. These open issues should be taken into account and drive future measurement-related research in the field.

## Figures and Tables

**Table 1:** Wireless positioning systems and corresponding measurement techniques.

System	Measurement Technique
GNSS (e.g., Galileo, GPS)	TDoA
A-GNSS	TDoA
WLAN	AoA/RSSI/ToA, radio fingerprinting
Cellular	Cell ID/E-OTD/OTDoA/U-TDoA

**Table 2:** Sources of uncertainty in GNSS pseudoranges measured by a receiver.

Source	Typical	Notes
	Uncertainty	
Satellite clock	±2 m	Can vary between different
		GNSS systems
Satellite ephemeris	up to ±2.5 m	Even with corrections
		from GNSS ground control
		system
Ionospheric activity	>>±5 m	Signals in GNSS bands may
		be significantly affected
Tropospheric activity	not relevant	Compensated by
		DGNSS or RTK
Multipath	±2 m	Caused by reflections
		and delay from
		ground infrastructures
		and vegetation
Receiver noise	±0.1 m	Thermal noise by receiver RF
Receiver clock	not relevant	Due to cheap oscillators
		onboard receivers,
		easily mitigated

**Table 3:** Summary of train positioning approaches and reported results.

Approach	Test Conditions	Reported Result
Augmented GNSS [30]	Emulated train ride and RTK ground truth	train positioning error < 2 m
Locally Augmented	Static OBU and 2 RS located along the track:	train positioning error in [−2÷4] m,
GNSS [44]	Leipzig (IGS), Zurich (EGNOS)	some spikes in [−5÷6.5] m
	Field test: 3 there and back travels (54.53 km covered),	train velocity error < 2 km/h, 93.53% prob. (no GNSS outages) < 1 km/h, 80.90% prob.
GNSS and 10 DOF IMU	variable time duration GNSS outages in tunnels,	
fusion [33]	reference velocity by tachometer and Doppler radar readings	train velocity error < 2 km/h, 88.90% prob. (with GNSS outages) < 1 km/h, 76.85% prob.
	Test-bed with GNSS visibility conditions:	
GNSS & 5G	clear-sky, urban, canyon environments.	best mean positioning accuracy
hybridization [38]	5G BS-UE locations: N20, (N20 E20), (N20 SE20),	at 99th percentile:
	N50, (N50 E50), (N50 SE50).	
	Single frequency GNSS (L1).	clear-sky: 0.320 m, in (N20 E20) PPP&5G
	Ground truth: geo-ref. position in open-sky;	urban: 0.918 m, in (N20 E20) 5G only
	precisely computed position	canyon: 0.7272 m, in (N20 E20) 5G only
	in urban and canyon environments	
5G only [43]	Simulated track of over 43 km	mean positioning accuracy: 0.66 m
	with variable train velocity of up to 400 km/h	95% errors < 1.7 m, 99% errors < 2.3 m

**Table 4:** LDS Accuracy requirements for rail.

Train LDS Functionality	Accuracy (95%)
VB detection (vital)	25 cm
VB detection (non-vital)	125 cm
Track discrimination	50 cm
